# Clinical Management of Bowel Dysfunction After Low Anterior Resection for Rectal Cancer

**Published:** 2016-09-01

**Authors:** Angela Bazzell, Lydia T. Madsen, Joyce Dains

**Affiliations:** The University of Texas MD Anderson Cancer Center, Houston, Texas

## Abstract

The American Cancer Society estimated that 39,610 new cases of rectal cancer were diagnosed in the United States in 2015. Surgery is the primary treatment for rectal cancer, with the majority of patients undergoing sphincter-preserving surgery with low anterior resection. Although low anterior resection can prevent patients from having a permanent colostomy, bowel dysfunction may occur in 60% to 90% of patients. Bowel dysfunction symptoms may include fecal and gas incontinence, urgency, frequent bowel movements, clustering of stools, and difficulty emptying. The symptoms collectively are referred to as low anterior resection syndrome (LARS) and adversely affect quality of life. There are no specific therapies for management of LARS. This comprehensive literature review evaluates evidence-based, clinical nonsurgical interventions for symptom management of LARS and will assist advanced practitioners in recognizing symptoms and implementing clinical interventions in the outpatient setting for management of LARS.

The American Cancer Society estimated that 39,610 new cases of rectal cancer were diagnosed in the United States in 2015 (Siegel, Miller, & Jemal, 2015). Current treatment options for rectal cancer include chemotherapy, radiation therapy, and surgery. The main goals in the treatment of rectal cancer include "local control; long-term survival; preservation of anal sphincter, bladder, and sexual function; and maintenance or improvement in quality of life" (Balch, De Meo, & Guillem, 2006).

Surgery is the most common treatment for rectal cancer and is based upon tumor location, preoperative staging, and the presence or absence of high-risk features (National Cancer Institute, 2015). For decades, abdominoperineal resection (APR), with removal of the anus, rectum, and part of the sigmoid colon with a permanent colostomy, was the standard surgery for rectal cancer. Increased knowledge of how rectal cancer spreads, improvement in surgical techniques, development of circular stapling devices, and neoadjuvant therapy have advanced the use of sphincter-preserving surgery with low anterior resection (LAR) as the preferred treatment for upper and middle rectal cancers (Pachler & Wille-Jørgensen, 2012). Low anterior resection includes removing the sigmoid colon and middle or upper rectum, with a low colorectal anastomosis to avoid a permanent colostomy (Emmertsen & Laurberg, 2012). Temple et al. (2009) found 77% of patients with stage I–III rectal cancers who receive care at National Comprehensive Cancer Network (NCCN)–designated institutions undergo sphincter-preserving surgery.

## SCOPE OF THE PROBLEM

Low anterior resection preserves the sphincter and avoids a permanent ostomy. Historically, patients with a permanent ostomy were thought to have a poorer quality of life than patients without a permanent ostomy. However, following LAR, studies have shown 60% to 90% of patients will experience some degree of bowel dysfunction or change in bowel habits (Bryant, Lunniss, Knowles, Thaha, & Chan, 2012; Juul et al., 2014a).

Bowel dysfunction after LAR is referred to as low anterior resection syndrome (LARS) and may consist of fecal and gas incontinence, urgency, frequent bowel movements, clustering of stools, and difficulty emptying (Chen, Emmertsen, & Laurberg, 2014; Juul et al., 2014b). Low anterior resection syndrome negatively affects quality of life by impacting emotional, physical, social, and role functioning (Juul et al., 2014b). The symptoms are generally more pronounced within the first 12 months postoperatively, stabilizing within the first 2 years after surgery (Emmertsen, Laurberg, & the Rectal Cancer Function Study Group, 2013). Symptoms may persist up to 15 years after anterior resection for some patients and may vary in severity (Bryant et al., 2012). Currently, there are no specific treatments for LARS, and standard symptom management with existing therapies, such as diphenoxylate hydrochloride and atropine sulfate (Lomotil), loperamide, and dietary changes, is empirical and symptom-based (Bryant et al., 2012).

From 2005 to 2009, the 5-year net survival for patients in the United States diagnosed with rectal cancer was 64% (Allemani et al., 2015). Given the prevalence of bowel dysfunction, the length of time symptoms continue postoperatively, the lack of specific therapies for LARS, and the negative impact on quality of life, advanced practitioners (APs) should be aware of the symptoms that matter to patients for identification and clinical management of LARS. A comprehensive review of the literature from 2009 to 2014 was conducted to evaluate current, evidence-based clinical nonsurgical interventions for symptom management of LARS.

## METHODS

A comprehensive review of the literature was conducted to gather peer-reviewed evidence on interventions that addressed symptoms of bowel dysfunction following LAR for rectal cancer.

Electronic databases searched included PubMed, Scopus, Ovid, Cochrane Library, and the Cumulative Index to Nursing and Allied Health Literature (CINAHL) with the following search terms applied: anterior resection AND rectal cancer AND fecal incontinence; anterior resection syndrome AND rectal cancer; low anterior resection syndrome; rectal cancer AND defecation AND anterior resection; anterior resection syndrome. The medical subject heading terms used were rectal surgery AND fecal incontinence. A research librarian assisted with the literature search. A secondary review of references for relevancy, in addition to systematic reviews or meta-analyses to identify additional primary sources, was included.

Multiple inclusion and exclusion criteria were applied. Articles published after January 1, 2009, and before December 31, 2014, in English, and related to studies of humans were included. Posters, abstracts, and oral abstracts were excluded. Studies addressing clinical management of bowel dysfunction following anterior resection for rectal cancer were included. Studies that focused on surgical techniques, surgical interventions, fecal incontinence, or bowel dysfunction not related to anterior resection; the role of neoadjuvant radiation therapy; and quality of life were beyond the scope of this review and were excluded.

A total of 160 full-text articles were initially identified, including 3 retrieved from the reference lists review. Of them, 152 were excluded based on the inclusion and exclusion criteria. Eight articles met the inclusion and exclusion criteria and are part of this review.

## RESULTS

The clinical nonsurgical interventions of biofeedback therapy with pelvic floor exercises, Kegel exercises with pelvic muscle strengthening, pharmacologic symptom management, and colonic and transanal irrigation, designed to improve the symptoms of LARS, met the inclusion and exclusion criteria and are included in this review. Intervention results are organized by the symptoms identified by patients as most impactful on quality of life: fecal and gas incontinence, frequency of bowel movements, clustering of stools, and urgency (Emmertsen & Laurberg, 2012). A summary of the results of these eight studies is presented in Table 1.

**Table 1:**
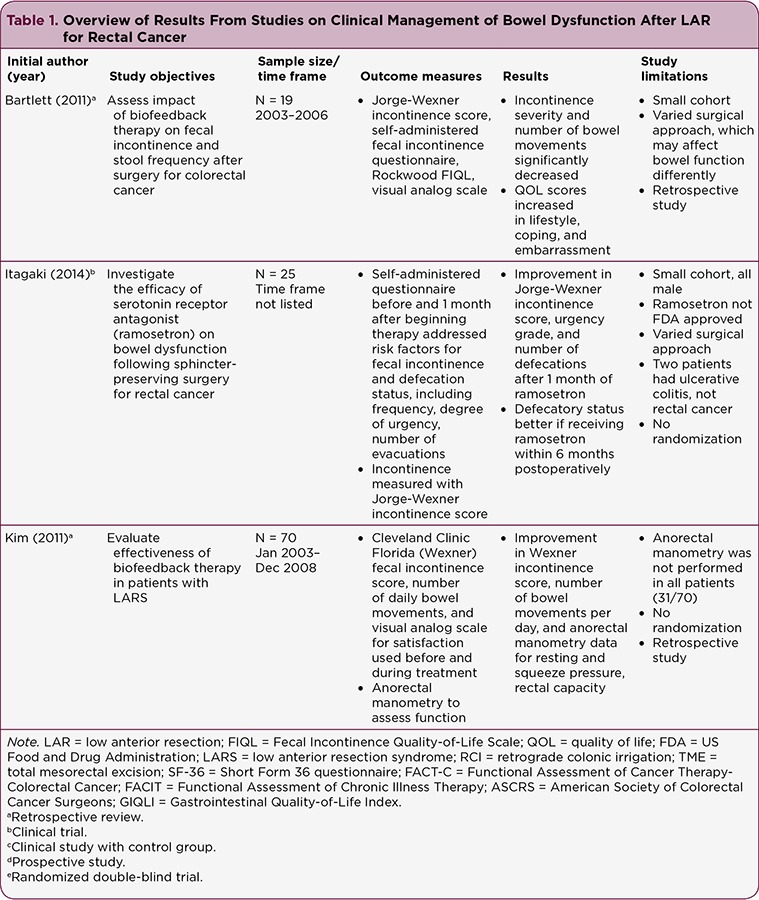
Overview of Results From Studies on Clinical Management of Bowel Dysfunction After LAR for Rectal Cancer

**Table 1:**
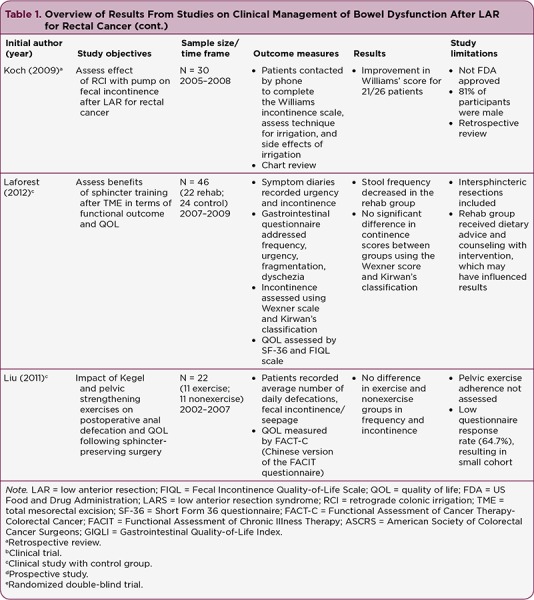
Overview of Results From Studies on Clinical Management of Bowel Dysfunction After LAR for Rectal Cancer (cont.)

**Table 1:**
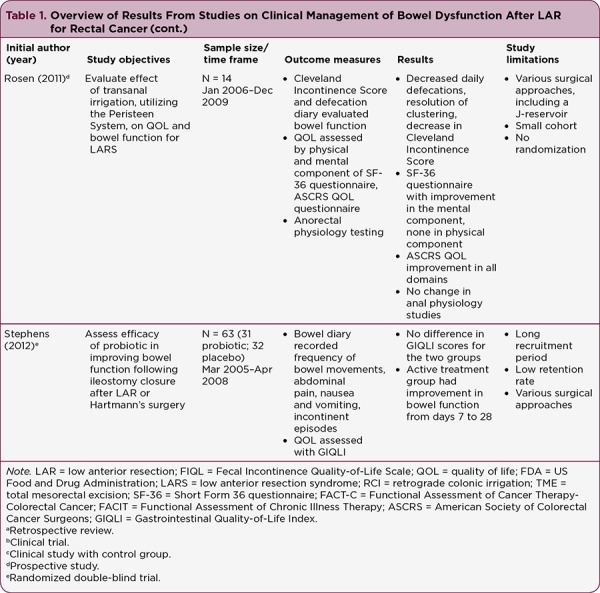
Overview of Results From Studies on Clinical Management of Bowel Dysfunction After LAR for Rectal Cancer (cont.)

**Fecal and Gas Incontinence**

Studies reviewed measured fecal and gas incontinence using different scales and methods. The Cleveland Clinic Florida Fecal Incontinence Score (CCFFIS; also known as the Jorge-Wexner incontinence score), Williams incontinence scale, Kirwan classification, and bowel diaries were used to evaluate incontinence. The CCFFIS, Williams incontinence scale, and Kirwan classification utilize questionnaires to assess the degree and frequency of incontinence to gas, liquid, and solid stool and provide a numeric score or grade based on the results (Jorge & Wexner, 1993; Williams, Patel, George, Hallan, & Watkins, 1991; Kirwan, Turnbull, Fazio, & Weakley, 1978). The CCFFIS also includes whether patients wear a pad or have altered their lifestyle as part of the continence score (Jorge & Wexner, 1993). Bowel diaries record the incidence of incontinence over a specified period.

*Biofeedback With Pelvic Floor Exercises*: Bartlett, Sloots, Nowak, and Ho (2011) addressed the symptoms of fecal and gas incontinence in a trial that included four to five outpatient treatment sessions of biofeedback therapy. These sessions consisted of visual and verbal feedback, anal sphincter and pelvic floor exercises, advice on coping strategies and diet, and individualized instructions for 4 weeks of daily home exercises. Incontinence was measured using the CCFFIS (Bartlett et al., 2011).

There were statistically significant improvements in participants’ incontinence score at the end of the sessions (*p* = .001; Bartlett et al., 2011). At 2-year follow-up, the investigators found incontinence scores in 12 of the 19 participants "marginally worsened… *p* > .05" (Bartlett et al., 2011, p. 323), with 25% of participants reporting they had forgotten how to do the exercises. However, although marginally worsened, the incontinence scores at follow-up remained better than baseline incontinence scores.

Kim et al. (2011) evaluated the effectiveness of biofeedback therapy involving coordination training, sensory training, and strength training, performed once weekly for 10 consecutive weeks. Electromyographic (EMG) activity was monitored, a home pelvic floor exercise program was provided, and participants were offered rectal balloon training as part of pelvic floor rehabilitation. Statistically significant improvements were found in CCFFIS incontinence scores (*p* < .001) following treatment (Kim et al., 2011). Participants demonstrated the greatest improvement in CCFFIS incontinence scores (*p* = .032) when biofeedback therapy and pelvic floor exercises were initiated more than 18 months after surgery (Kim et al., 2011).

Laforest et al. (2012) also used biofeedback therapy with pelvic floor exercises as an intervention, with participants in the rehabilitation group (n = 22) attending 15 one-hour training sessions, once per week. The intervention included initial EMG assessment, biofeedback exercises, instruction on pelvic floor exercises, and visual feedback with rectal balloon training (Laforest et al., 2012). However, in this study, no significant difference was found in the Jorge-Wexner incontinence score (*p* = .10) and Kirwan classification (*p* = 1.00) between the rehabilitation group (n = 22) and control group (n = 24).

*Kegel Exercises With Pelvic Muscle Strengthening*: Liu, Chen, and Lee (2011) reported on pelvic strengthening and Kegel exercises, which did not improve incontinence between the exercise (n = 11) and nonexercise group (n = 11; p = 1.000). Participants were provided handouts and demonstrations of Kegel exercises, with instructions to perform the exercises for 10 minutes, three to four times per day, along with exercises to strengthen the pelvic muscles. Incontinence was assessed by participants self-reporting fecal incontinence or fecal seepage. To evaluate the long-term use of Kegel exercises, Liu et al. (2011) followed up with 15 patients 2 years post colostomy closure and found there was no significant difference in continence between the exercise (n = 4) and nonexercise group (n = 11; p = .569).

*Pharmacology*: Itagaki et al. (2014) as well as Stephens and Hewett (2012) evaluated the efficacy of pharmacologic symptom management in improving incontinence with LARS. However, only Itagaki et al. (2014) reported incontinence as an outcome measure, noting a statistically significant difference in CCFFIS after participants received the oral serotonin (5-HT3 [serotonin]) receptor antagonist ramosetron once daily for 1 month (*p* < .01). Notably, no difference in incontinence scores between participants given ramosetron within 6 months of surgery (n = 16) and participants who received ramosetron more than 6 months postoperatively (n = 9) was observed (Itagaki et al., 2014).

Stephens and Hewett (2012) conducted a double-blind, placebo-controlled, randomized trial of the probiotic VSL#3 in the immediate postoperative period following ileostomy closure. The treatment group (n = 31) was given the probiotic twice daily for 4 weeks, and the nontreatment group (n = 32) received placebo. Participants completed a bowel function diary, including reporting incontinent episodes during the treatment period. However, the statistical outcomes of the intervention on incontinence were not reported in the study results.

*Colonic and Transanal Irrigation*: Koch, Rietveld, Govaert, van Gemert, and Baeten (2009) as well as Rosen, Robert-Yap, Tentschert, Lechner, and Roche (2011) showed significantly improved incontinence scores with interventions that used colonic and transanal irrigation for symptom management of LARS.

Koch et al. (2009) evaluated the effect of daily retrograde colonic irrigation (RCI) on fecal incontinence following LAR. Fecal incontinence was measured using the Williams incontinence score. In the study, RCI was performed with an irrigation pump, using a flexible tube introduced into the anal canal, irrigating up to 500 mL of body temperature water. Patients who were selected to receive an irrigation pump for fecal incontinence between 2005 and 2008 were interviewed by phone and a questionnaire completed; additional data were collected by chart review. There was a statistically significant improvement in incontinence scores (*p* < .0001) in the 21 patients (n = 26) who continued to perform RCI at the time of the study (Koch et al., 2009).

The effect of transanal irrigation (TAI) on bowel dysfunction using the Peristeen® anal irrigation system following anterior resection was reported by Rosen et al. (2011). A trained stoma nurse instructed participants on how to insert a rectal catheter with a retaining balloon and irrigate up to 1,500 mL of lukewarm tap water into the rectum. The investigators reported a statistically significant improvement in participant CCFFIS incontinence scores (*p* < .01) at last follow-up, with a median time using TAI of 29 months (range, 15–46 months).

Bartlett et al. (2011), Koch et al. (2009), and Laforest et al. (2012) addressed incontinence to flatus specifically. Bartlett et al. (2011) found participants who completed biofeedback therapy with pelvic floor exercises (n = 19) had a statistically significant reduction in flatus incontinence score (*p* = .017). Of the patients included in the data analysis for RCI, three (14.2%, n = 21) reported incontinence of flatus after initiation of RCI (Koch et al., 2009). Baseline information for the number of patients with incontinence of flatus prior to starting RCI was not reported, however. Laforest et al. (2012) reported no significant difference (*p* = 1.00) related to incontinence of flatus between the rehabilitation group (n = 22) and the control group (n = 24) after undergoing biofeedback therapy with pelvic floor exercises.

**Frequency of Bowel Movements**

*Biofeedback Therapy With Pelvic Floor Exercises*: Bartlett et al. (2011), Kim et al. (2011), and Laforest et al. (2012) showed significant improvement in stool frequency when assessing the impact of biofeedback therapy and pelvic floor exercise on LARS. Bartlett et al. (2011) found a significant reduction in stool frequency (*p* = .003) as well as a "marginally improved" stool consistency, as measured by the Bristol Stool Scale. Kim et al. (2011) showed that participants had a significant decrease (*p* < .001) in the number of daily bowel movements. Participants who presented with frequent defecation or fecal incontinence as the primary symptom showed a significant decrease in bowel frequency (*p* = .019; p < .001), compared with patients who presented with incomplete evacuation as the primary symptom (*p* = .321). Laforest et al. (2012) reported stool frequency was significantly lower (*p* = .025) in the rehabilitation group (n = 22) than the control group (n = 24). However, comparative data were not reported on stool frequency between the two groups prior to initiation of therapy.

*Kegel Exercises with Pelvic Muscle Strengthening*: Liu et al. (2011) reported that pelvic floor and Kegel exercises, instituted in the exercise group postoperatively (n = 11), did not improve the frequency of defecation. There was no statistical difference (*p* = 1.00) between the exercise group and the nonexercise group related to average daily bowel frequency. The study also noted that no statistical difference (*p* = 1.00) existed between the exercise group (n = 11) and the nonexercise group (n = 11) 2 years post colostomy closure.

*Pharmacology*: Both of the studies evaluating pharmacologic symptom management of LARS showed improvement, if not statistical significance, in bowel frequency (Itagaki et al., 2014; Stephens & Hewett, 2012).

Itagaki et al. (2014) reported that the number of defecations per day decreased significantly (*p* < .01) after 1 month of daily ramosetron. The study found daily frequency was significantly improved in participants prescribed ramosetron within 6 months postoperatively (*p* < .01) compared with participants who had not received ramosetron at more than 6 months postoperatively (*p* < .050).

Stephens and Hewett (2012) found that all participants who completed the study on the efficacy of VSL#3 for LARS improved in the number of daily bowel movements. There was statistically significant improvement in bowel frequency in the treatment group, from day 7 to day 28 postoperatively (*p* = .48). However, the investigators also reported there was no statistical difference in the frequency of bowel movements experienced by the placebo group (n = 32) or the treatment group (n = 31) at the end of each week of treatment, concluding VSL#3 was not effective in improving bowel function.

*Transanal Irrigation*: Rosen et al. (2011) evaluated TAI in the treatment of LARS and reported frequency as an outcome measure, noting that the number of daily defecations decreased significantly (*p* < .001) with TAI; "the majority of patients were able to empty their bowels with one to two defecations over 24 to 48 hours."

**Clustering of Stools**

*Biofeedback Therapy With Pelvic Floor Exercises*: Clustering of stools refers to numerous bowel movements occurring within a short period. Laforest et al. (2012) defined "stool fragmentation" as two or more bowel movements within 1 hour. After undergoing biofeedback exercises with pelvic floor exercises, 65% of participants continued to experience clustering, or stool fragmentation, with no significant difference (*p* = .50) between the rehabilitation group (n = 22) and the control group (n = 24). There were no numerical data documenting the number of participants experiencing clustering prior to initiating therapy, but Laforest et al. (2012) did report stool fragmentation data in the functional results, which included a control group.

*Transanal Irrigation*: Rosen et al. (2011) did not define clustering but observed that all patients (n = 14) prior to entry into the study "complained of clustering with urgency." Patients reported no clustering after TAI was established for a median of 29 months (range, 15–46 months).

**Urgency**

*Biofeedback Therapy With Pelvic Floor Exercises*: Laforest et al. (2012) defined urgency as "the ability to defer stool evacuation for more than 15 minutes." The researchers found no significant difference (*p* = 1.00) in urgency for patients who underwent the previously described biofeedback therapy with pelvic floor exercises (n = 22), compared with those in the control group (n = 24).

*Pharmacology*: Itagaki et al. (2014) categorized the degree of urgency from 0–3, with a score of 0 as no urgency, signifying participants could always defer defecation for 10 minutes, and a score of 3 as severe urgency, signifying participants could never defer defecation for 10 minutes, more than once daily. The investigators reported patients’ urgency grade decreased from 2.3 to 1.2 (*p* < .01) after taking ramosetron for 1 month, and the urgency grade of patients who received ramosetron within 6 months of surgery was significantly improved (*p* < .01) compared with patients who received ramosetron more than 6 months after surgery (*p* < 0.05).

## DISCUSSION

Four of the eight studies provided evidence for clinical management interventions designed to decrease the symptoms of incontinence and stool frequency following LAR (Bartlett et al., 2011; Itagaki et al., 2014; Kim et al., 2011; Rosen et al., 2011). Koch et al. (2009) reported significant improvement in continence scores but did not address participant stool frequency in the results. Laforest et al. (2012) found no significant difference in continence between the rehabilitation and control groups but found stool frequency decreased significantly in the rehabilitation group. Studies evaluating probiotic use and implementing Kegel exercises and pelvic muscle strengthening postoperatively were not associated with statistically significant improvement in stool frequency or incontinence (Liu et al., 2011; Stephens & Hewett, 2012).

With the exception of the study by Stephens and Hewett (2012), all studies addressed incontinence as an outcome measure, providing either an incontinence score or evaluating the frequency of incontinence. Bartlett et al. (2011), Itagaki et al. (2014), Kim et al. (2011), Koch et al. (2009), and Rosen et al. (2011) reported statistically significant improvement in incontinence following intervention, although Laforest et al. (2012) and Liu et al. (2011) reported no significant difference in participant incontinence.

**Study Limitations and Comparative Challenges**

One of the limitations in the studies by Itagaki et al. (2014) and Koch et al. (2009) is the lack of FDA approval for the interventions under evaluation. Itagaki et al. (2014) evaluated the efficacy of ramosetron in LARS, which is not approved by the US Food and Drug Administration (FDA). Ramosetron is approved in Japan (as Irribow) for diarrhea-predominant irritable bowel syndrome (IBS-D) in males, and all participants in the study were male (Itagaki et al., 2014). Alosetron (Lotronex) is the only FDA-approved 5-HT3 receptor antagonist for IBS-D and is approved for women with severe IBS-D, with restrictive use (Camilleri, 2013). Similar to ramosetron, the Biotrol® Irrimatic pump utilized for colonic irrigation in the Koch et al. (2009) study has not been approved by the FDA.

Itagaki et al. (2014) also included patients who had undergone different types of surgeries for differing diagnoses. Of the 25 participants, 23 had undergone surgery for rectal cancer, and 2 had undergone surgery for ulcerative colitis. The Laforest et al. (2012) intervention included only participants who had undergone sphincter-sparing surgery for rectal cancer.

Stephens and Hewett (2012) were limited by the high number of patients who withdrew from the study. A total of 38 patients (62.29%) completed the study, increasing the likelihood of a statistical type 2 error in which the study was underpowered and the impact was undetected.

An obstacle to the data synthesis was that no single assessment tool for fecal and gas incontinence was consistently used in the studies included in this review. Liu et al. (2011) as well as Stephens and Hewett (2012) utilized bowel diaries to assess bowel function. The CCFFIS was used most frequently as an outcome measure for incontinence (Table 2). Five studies—Bartlett et al. (2011), Itagaki et al. (2014), Kim et al. (2011), Laforest et al. (2012), and Rosen et al. (2011)—used the CCFFIS. The Williams incontinence scale and Kirwan classification were also used to evaluate incontinence. Reporting the CCFFIS, Williams score, and Kirwan’s classification as an outcome measure does not distinguish between incontinence to gas, liquid, or solid stool, making it impossible to evaluate the effect of the interventions on the symptoms of gas incontinence and fecal incontinence independently.

**Table 2:**
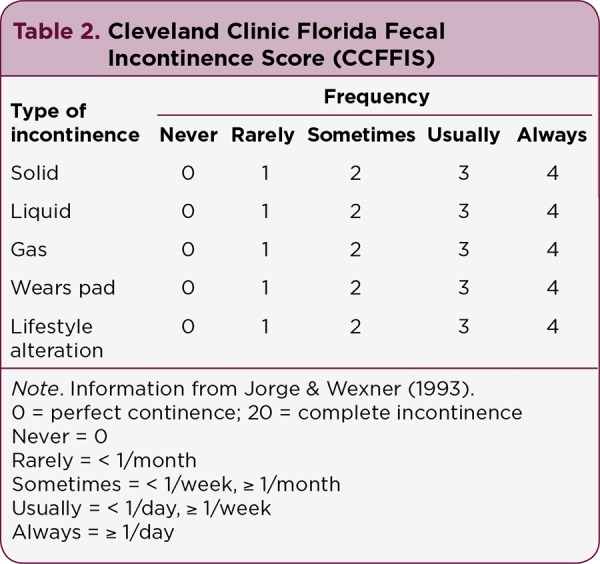
Cleveland Clinic Florida Fecal Incontinence Score (CCFFIS)

Interventions that targeted symptoms of clustering and urgency were not found; only two studies reported a decrease in either symptom. Transanal irrigation positively impacted the frequency of clustering following initiation of regular therapy, and patients reported decreased urgency after 1 month of ramosetron (Itagaki et al., 2014; Rosen et al., 2011). The lack of interventions directed at clustering and urgency related to LARS may be attributed to providers’ lack of knowledge of the impact of these variables on patient quality of life and frequency of occurrence. Chen et al. (2014) found that 1 of 58 rectal cancer specialists (colorectal surgeons and radiation oncologists) were able to correctly identify all 5 symptoms on the LARS score questionnaire, overestimating the impact of fecal incontinence and frequency and underestimating the impact of clustering and urgency.

Of the eight interventions reviewed, none addressed or improved all symptoms identified by patients as most impactful to quality of life (Table 3). Many of the studies included in this review were also retrospective studies, with bowel diaries and questionnaires requiring patient recall, which can lead to observation bias. Underreporting of the severity of symptoms by participants, perhaps due to embarrassment, may have also occurred. Laforest et al. (2012) and Liu et al. (2011) did not measure variables before and after interventions, making comparison a challenge. The trial by Stephens and Hewett (2012) was the sole randomized double-blind study. The lack of randomized clinical trials along with the lack of a standardized symptom assessment tool makes it difficult to compare study outcomes.

**Table 3:**
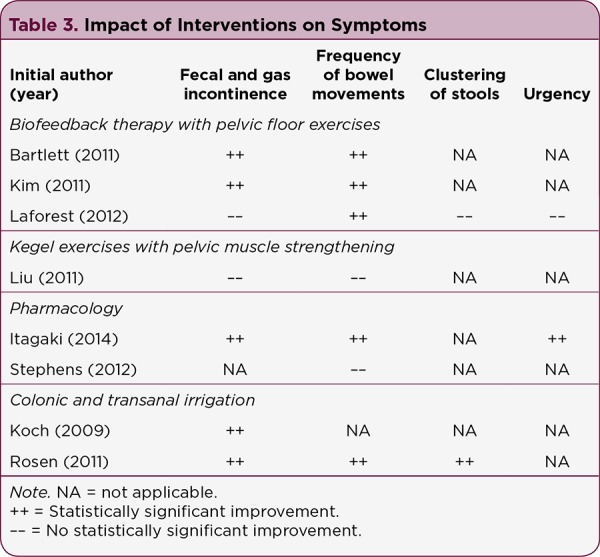
Impact of Interventions on Symptoms

The studies reviewed were limited by the small number of participants (n = 14–70) and the use of single institutions. Rosen et al. (2011) as well as Stephens and Hewett (2012) were the only studies conducted at more than one institution. Differing surgery criteria were also used in several studies. Kim et al. (2011), Koch et al. (2009), Liu et al. (2011), and Rosen et al. (2011) included only patients who had undergone sphincter-preserving surgeries. Other studies included patients who had undergone total proctocolectomy, reversal of Hartmann’s procedure, and segmental colectomy. Itagaki et al. (2014) also included patients with ulcerative colitis. The small number of participants at single institutions, with differing surgical procedures and diagnoses, may also limit the ability to draw conclusions on the effectiveness of the interventions.

## FUTURE RESEARCH

Analyzing postoperative symptom data has been limited by the lack of a simple, uniform assessment tool to identify the most bothersome symptoms of bowel dysfunction for patients, which can influence quality of life. Available assessment tools vary widely and often evaluate the incidence of symptoms and quality of life as separate constructs or focus on fecal incontinence without assessing other symptoms associated with LARS (Emmertsen & Laurberg, 2012).

To evaluate the impact of symptoms following LAR on quality of life, Emmertsen and Laurberg (2012) developed a scoring system for bowel dysfunction based on a cohort of 961 Danish patients who underwent LAR for rectal cancer between 2001 and 2007. The LARS score utilizes a five-item, self-administered questionnaire and measures the five most important symptoms identified by patients (i.e., "incontinence for flatus, incontinence for liquid stool, frequency of bowel movements, clustering of stools, and urgency" (Table 4). The symptoms are assigned a score value based on the correlation with their impact on quality of life, with a score range of 0 to 42. The LARS score was validated internationally by Juul et al. (2014a).

**Table 4:**
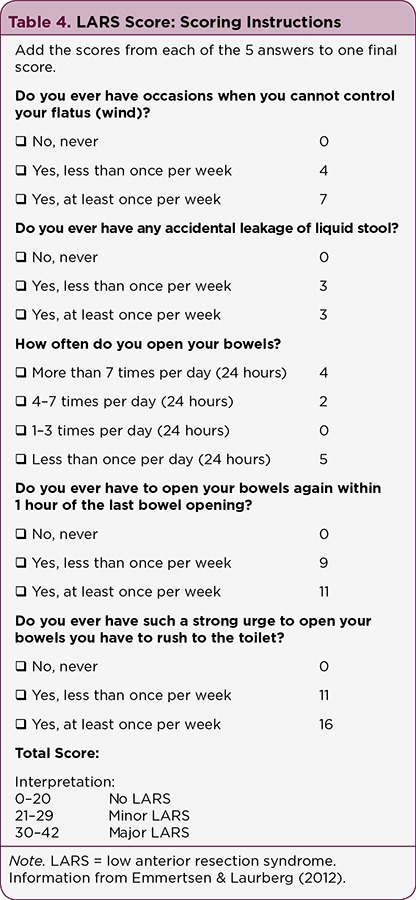
LARS Score: Scoring Instructions

Utilization of the LARS score in clinical practice will help identify patients with LARS and assist in evaluating the effect of management interventions on bowel dysfunction symptoms. Use of a validated questionnaire in clinical research will also allow a consistent scoring system to capture data related to frequency of symptoms and compare outcomes of interventions. An assessment tool that advances research and assists in communication between patients and APs may lead to improvement in patients’ quality of life.

## CONCLUSION

In a 2012 Cochrane review, Pachler and Wille-Jørgensen (2012) compared quality of life in patients with rectal cancer, with or without a permanent colostomy, finding that quality of life after anterior resection was not superior to that with a permanent colostomy. The review included 35 studies in which quality of life was measured using a validated quality-of-life instrument, in patients with rectal cancer treated with APR, Hartmann’s operation, or LAR. A total of 14 studies found patients with a permanent ostomy did not have a poorer quality of life, whereas the rest of the studies found some difference between the two groups, not always favoring patients without an ostomy.

This review identifies the importance of discussing bowel function with patients before surgery for rectal cancer, as the etiology of LARS is likely related to multiple factors and may require a multifactorial and/or multidisciplinary approach to postoperative symptom management. Patients should receive detailed preoperative education regarding the risk of significant postoperative bowel dysfunction and the efficacy of available nonsurgical interventions to improve bowel dysfunction. Future clinical research should also be directed at identifying an effective combination of clinical interventions that provides the best symptom control for patients based on their individual symptoms.

The degree of bowel dysfunction following LAR for rectal cancer and the lack of conservative, well-studied interventions for symptom management pose a significant challenge to APs. Communicating with patients before and after surgery as well as utilizing a validated assessment tool, such as the LARS score, will help direct therapies at symptoms that most impact a patient’s life.
